# From Ingestion to Septic Shock: A Case Report on Esophageal Perforation and Mediastinitis Following Foreign Body Ingestion

**DOI:** 10.7759/cureus.105183

**Published:** 2026-03-13

**Authors:** Aimad Ahmed Khamlij, Karim Hdach, Ali Habbal, Fouzia Douirek, Amra Ziadi

**Affiliations:** 1 Surgical Intensive Care Unit B/Anesthesia and Critical Care, University Hospital Mohammed VI/Cadi Ayyad University, Marrakech, MAR

**Keywords:** esophageal perforation, foreign body, mediastinitis, morocco, septic shock

## Abstract

Acute mediastinitis is a rare but life-threatening condition, most commonly secondary to esophageal perforation. Foreign body ingestion in adults is an unusual etiology, with limited data from Morocco, where only a few cases have been reported. We report a case of mediastinitis complicating esophageal perforation following accidental foreign body ingestion in a Moroccan patient, highlighting diagnostic challenges and management in a resource-limited setting. We report the case of a 38-year-old patient admitted with septic shock, complicated by dysphagia and epigastric pain, five days after accidental foreign body ingestion. A cervicothoracic CT scan revealed esophageal perforation complicated by mediastinitis and inhalation pneumonia. Emergency surgical management consisted of a Kocher cervicotomy with mediastinal drainage, combined with early broad-spectrum antibiotic therapy, which led to a favorable postoperative outcome. This case from Morocco underscores the importance of early clinical suspicion, prompt CT imaging, and aggressive medico-surgical management in improving outcomes, even in delayed presentations. It contributes to the growing body of regional literature on foreign body-induced mediastinitis in North Africa.

## Introduction

Acute mediastinitis is a life-threatening infection of the mediastinum, most commonly secondary to esophageal perforation. The latter can be iatrogenic, traumatic, or, more rarely, related to foreign body ingestion [1]. Although the majority of ingested foreign bodies pass spontaneously through the digestive tract, their esophageal impaction exposes patients to severe complications, notably perforation and mediastinitis [2].

Diagnostic delay, favored by often non-specific initial symptoms, is the main factor contributing to a poor prognosis. We report a case of septic shock secondary to acute mediastinitis caused by esophageal perforation following accidental foreign body ingestion in a Moroccan patient.

Through this observation, we aim to highlight the diagnostic challenges in our setting, describe a successful management strategy despite delayed presentation, and contribute to the growing body of regional literature on this rare but serious condition.

## Case presentation

**Figure 1:**
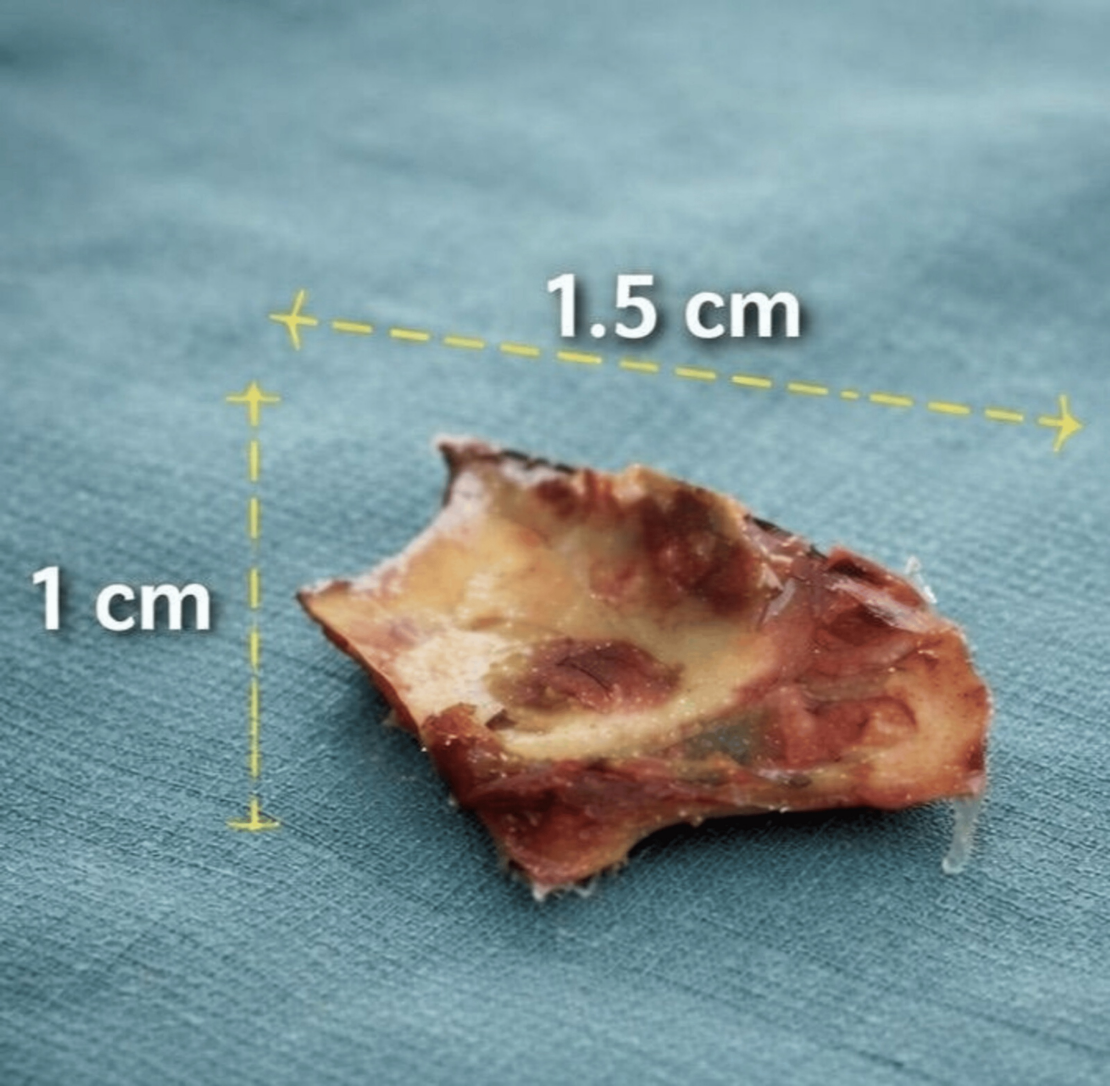
Foreign body (chicken bone)

A 38-year-old male, a chronic smoker with a 14-pack-year history, was admitted for the management of mediastinitis secondary to esophageal perforation, complicated by septic shock, following the accidental ingestion of a foreign body (chicken bone) (Figure 1).

The patient accidentally ingested a chicken bone five days prior to admission. Following ingestion, he progressively developed dysphagia for both solids and liquids, associated with epigastric pain and a productive cough. These symptoms evolved over the five days in a context of altered general condition, with no reported fever, dyspnea, or acute chest pain. He presented to the emergency department on day 5 with septic shock.

On admission, the patient was confused and hemodynamically unstable, with a blood pressure of 85/35 mmHg and a heart rate of 132 beats per minute. At presentation, he had a respiratory rate of 25 cycles per minute and an oxygen saturation of 88% on room air. 

**Figure 2:**
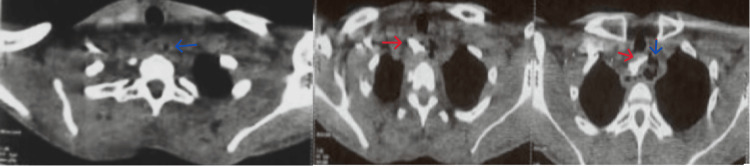
Cervicothoracic CT scan Axial cervicothoracic CT scan showing mediastinal air and fat infiltration( blue arrow), foreign body (red arrow)

A cervicothoracic CT scan revealed diffuse thickening of the esophageal wall, predominantly in the upper third, associated with the presence of multiple mediastinal air bubbles, marked infiltration of mediastinal fat, and multiple mediastinal lymphadenopathies, all findings suggestive of esophageal perforation complicated by acute mediastinitis. Furthermore, an area of right paramediastinal pulmonary consolidation was noted, associated with adjacent ground-glass opacities and regular thickening of the septal lines, suggesting bilateral inhalation pneumonia (Figure 2).

The initial laboratory workup revealed a severe inflammatory and infectious syndrome. There was hyperleukocytosis at 13,590/mm³ with neutrophilic predominance (absolute neutrophil count (ANC) 11,800/mm³), associated with a major elevation of C-reactive protein to 329 mg/L. Hemoglobin was 12.9 g/dL, and platelet count was 258,000/mm³. Lactate was 3 mmol/L, with a pH of 7.22. Electrolyte panel showed sodium 135 mmol/L, potassium 4.8 mmol/L, and chloride 103 mmol/L, with no major electrolyte disorder. Renal function was preserved: urea 1.92 mmol/L and creatinine 49.3 µmol/L.

Initial management consisted of stabilization with invasive monitoring, placement of a central venous catheter, fluid resuscitation with 20 cc/kg of Ringer's lactate and norepinephrine, associated with an initial dose of broad-spectrum probabilistic antibiotic therapy combining piperacillin-tazobactam 4 g and vancomycin 2 g over 2 hours. After orotracheal intubation and mechanical ventilation, the patient underwent a Kocher cervicotomy with extraction of a chicken bone (Figure 1), primary closure of the shredded esophageal wall, protection of the suture line by the sternal head of the sternocleidomastoid muscle, and mediastinal drainage. A feeding jejunostomy was performed; no intraoperative bacteriological samples were collected, as no purulent collection was identified during surgical exploration.

Following surgery, the patient was admitted to the intensive care unit for postoperative management, where respiratory (ventilation) and hemodynamic support continued alongside antibiotic therapy with piperacillin-tazobactam (4 g × 3/day) and vancomycin (2 g/24 h). The patient was successfully extubated after 48 hours of ICU hospitalization.

The total length of stay in the intensive care unit was seven days, after which the patient was transferred to the thoracic surgery department. Oral liquid feeding was initiated two months postoperatively and was well-tolerated. Jejunostomy removal was planned at four months.

## Discussion

Acute mediastinitis secondary to esophageal perforation remains a rare but extremely serious pathology, associated with high mortality rates reported between 20 and 40% in various series, particularly when diagnosis is delayed [3,4]. Our observation illustrates this severity, with an initial presentation already complicated by septic shock, a situation described in advanced forms of mediastinitis. As reported by Brinster et al. and Kiernan et al., the initial severity strongly conditions the prognosis, regardless of the etiology [3,4].

In the benchmark series by Brinster et al., esophageal perforations are predominantly iatrogenic (59%), while perforations secondary to foreign body ingestion represent a minority of cases, estimated between 9% and 14% [3].

In Morocco, only a few similar cases have been previously documented. Merzouqi et al. reported a case of cervicomediastinal cellulitis in a 50-year-old woman who presented 9 days after chicken bone ingestion, requiring surgical drainage and an esophageal suture. In their case, *Streptococcus β-hemolytic* was isolated from pus cultures, allowing targeted antibiotic therapy [5]. Ziad et al. described a 21-year-old patient with retropharyngeal abscess and mediastinitis following the migration of a metallic foreign body, successfully managed by cervicotomy and drainage [6]. Our case adds to these regional observations, presenting a patient with septic shock at admission and a favorable outcome despite the absence of intraoperative bacteriological samples.

The upper third esophageal location observed in our patient is consistent with literature data, which describes a predilection for foreign body impaction in this anatomical region [3-7]. The absence of an esophageal serosa facilitates rapid dissemination of infection into the mediastinum, explaining the frequency of severe septic complications [4].

Diagnostic delay constitutes the main unfavorable prognostic factor. While Brinster et al. report a clear increase in mortality beyond 24 hours [3], the estimated five-day delay in our case theoretically placed the patient in a high-risk situation for progression, which is consistent with the occurrence of septic shock upon admission.

The clinical presentation of esophageal perforation is often misleading. Chest pain is the most common presenting symptom (70% to 90%). Additional early manifestations include difficulty swallowing or painful swallowing. As the condition progresses, patients may develop fever, rapid heart rate, pleural effusion, cough, low blood pressure, and respiratory failure, which can ultimately lead to septic shock [8]. Similar to observations reported by Eroglu et al., our patient presented with neither acute chest pain nor initial fever, despite severe mediastinal involvement, underscoring the non-specific nature of initial clinical signs in nearly 30% of cases [9].

A cervicothoracic CT scan is unanimously recognized as the reference examination, not only for confirming the diagnosis but also for guiding therapeutic management [10]. The signs found in our patient were mediastinal air, fat infiltration, mediastinal lymph nodes, and associated pulmonary complications.

Standard surgical management of esophageal perforation typically involves several key steps: debridement of devitalized tissue around the perforation, primary closure, which may be reinforced with a muscle or intercostal flap, along with thorough mediastinal and pleural debridement and drainage. When prolonged nutritional support is anticipated, a feeding gastrostomy or jejunostomy is often associated [7]. The approach used in our patient was consistent with these established principles.

Unlike some series where sepsis occurs secondarily, our patient presented with septic shock from the outset, reflecting advanced infectious dissemination [11].

The biological abnormalities observed in our case (leukocytosis, very high CRP, hyperlactatemia) are comparable to those described in severe forms of mediastinitis reported in the literature, with lactate elevation constituting a major prognostic marker of mortality [11]. The early use of norepinephrine, recommended as the first-line vasopressor by the Surviving Sepsis Campaign, is consistent with international standards and aligns with data from Rhodes et al. [12]

Regarding antibiotic therapy: No intraoperative bacteriological samples were obtained in our case, which represents a limitation. Consequently, antibiotic therapy remained empirical throughout the course, combining piperacillin-tazobactam and vancomycin to cover the oropharyngeal aerobic and anaerobic flora typically involved in post-esophageal perforation mediastinitis, in a patient who presented with septic shock [1-13]. This empirical strategy, although undocumented microbiologically, resulted in a favorable outcome, highlighting the potential efficacy of early, broad-spectrum antibiotic therapy aligned with guidelines, even in the absence of microbiological documentation.

Despite a diagnostic delay and initial septic shock, the favorable outcome observed in our case contrasts with the high mortality rates reported in the literature [3,4]. This evolution can be explained by several factors already identified as favorable prognostic indicators in large series: rapid and effective surgical control of the infectious focus [1], early resuscitation management in accordance with Sepsis-3 guidelines [11], promptly adapted antibiotic therapy based on microbiological data, and the patient's young age and absence of major comorbidities [9-13].

## Conclusions

Post-foreign body ingestion mediastinitis in adults is a rare but serious pathology. This case from Morocco is, to the best of our knowledge, the first reported case presenting with septic shock. It underscores the importance of a high index of suspicion in any patient with persistent dysphagia, particularly in suggestive circumstances, even in the absence of typical symptoms. While these observations cannot be generalized, they highlight the potential value of early CT imaging, adequate surgical management, broad-spectrum antibiotic therapy, and hemodynamic optimization in similar clinical situations, especially in resource-limited settings where delayed presentation is common.
